# Evaluation of sequence alignments and oligonucleotide probes with respect to three-dimensional structure of ribosomal RNA using ARB software package

**DOI:** 10.1186/1471-2105-7-240

**Published:** 2006-05-04

**Authors:** Yadhu Kumar, Ralf Westram, Peter Kipfer, Harald Meier, Wolfgang Ludwig

**Affiliations:** 1 Lehrstuhl für Mikrobiologie, Technische Universität München, D-85350 Freising, Germany; 2 Lehrstuhl für Computer Graphik und Visualisierung, Technische Universität München, D-85748 Garching, Germany; 3 Lehrstuhl für Rechnertechnik und Rechnerorganisation, Technische Universität München, D-85748 Garching, Germany

## Abstract

**Background:**

Availability of high-resolution RNA crystal structures for the 30S and 50S ribosomal subunits and the subsequent validation of comparative secondary structure models have prompted the biologists to use three-dimensional structure of ribosomal RNA (rRNA) for evaluating sequence alignments of rRNA genes. Furthermore, the secondary and tertiary structural features of rRNA are highly useful and successfully employed in designing rRNA targeted oligonucleotide probes intended for *in situ *hybridization experiments. RNA3D, a program to combine sequence alignment information with three-dimensional structure of rRNA was developed. Integration into ARB software package, which is used extensively by the scientific community for phylogenetic analysis and molecular probe designing, has substantially extended the functionality of ARB software suite with 3D environment.

**Results:**

Three-dimensional structure of rRNA is visualized in OpenGL 3D environment with the abilities to change the display and overlay information onto the molecule, dynamically. Phylogenetic information derived from the multiple sequence alignments can be overlaid onto the molecule structure in a real time. Superimposition of both statistical and non-statistical sequence associated information onto the rRNA 3D structure can be done using customizable color scheme, which is also applied to a textual sequence alignment for reference. Oligonucleotide probes designed by ARB probe design tools can be mapped onto the 3D structure along with the probe accessibility models for evaluation with respect to secondary and tertiary structural conformations of rRNA.

**Conclusion:**

Visualization of three-dimensional structure of rRNA in an intuitive display provides the biologists with the greater possibilities to carry out structure based phylogenetic analysis. Coupled with secondary structure models of rRNA, RNA3D program aids in validating the sequence alignments of rRNA genes and evaluating probe target sites. Superimposition of the information derived from the multiple sequence alignment onto the molecule dynamically allows the researchers to observe any sequence inherited characteristics (phylogenetic information) in real-time environment. The extended ARB software package is made freely available for the scientific community via .

## Background

The backbone of the modern taxonomy of the prokaryotes is almost exclusively based upon a phylogenetic network derived from comparative sequence analysis of the small subunit ribosomal RNA (rRNA) and the respective phylogenetic marker genes [1]. Since the function of rRNA is largely determined by its structure [2] and the general structure of rRNA is universally conserved across all the taxa that have been examined [3,4], the structural features of rRNA, even when not universally identical across the taxa, is more highly conserved than are the nucleotides. As more knowledge is gained with respect to rRNA higher order structure through the availability of thousands of SSU rRNA [5] and LSU rRNA [6] sequences, has led to a breakthrough in the insight into evolutionary relationships between bacterial phyla [4] and between the major eukaryotic kingdoms and protist taxa [7]. The unique properties of rRNA demonstrate that the evolution of rRNA genes must be considered based on the structural constraints.

The most basic principle of phylogenetic studies is only the homologous characters can provide meaningful markers of genealogical descent. So, clearly the accuracy of a phylogeny from molecular data is critically dependent on the accuracy of sequence alignment. When there is a significant variability between the sequences due to insertions, deletions and mutations occurred during the course of evolution, the alignment of such sequences becomes more difficult and problematic. Given that the number and character of positional differences between the aligned sequences are the basis for the inference of relationship, the primary alignments must be evaluated against certain criteria before processing with the treeing algorithms in order to reduce such ambiguities. In support, studies have revealed that even small differences in the sequence alignment can result in quite different phylogenies [8,9]. So, by using structural features of rRNA to "anchor" homologous positions, many of the inherent problems of aligning rRNA sequences can be reduced. Furthermore, not all the aligned nucleotide positions or all types of substitution changes can be treated equally in terms of phylogenetic relevance because the nucleotides within the rRNA molecule are involved in different kinds of interactions, including both hydrogen bonding to other nucleotides within the molecule and interactions with ribosomal proteins and other RNA molecules (transfer RNAs). Therefore the knowledge of structural motifs exhibited by rRNA is greatly useful to align and compare rRNA sequences in order to produce more accurate and biologically meaningful alignments of rRNA genes.

The comparative analysis of thousands of rRNA sequences has yielded more reliable RNA structure models [10], which are well established and routinely used in the structure based phylogenetic studies. And with the availability of high-resolution RNA crystal structures for the 30S [11] and 50S [12] ribosomal subunits and the subsequent validation of comparative rRNA secondary structure models [10], the biologists are impelled to use three-dimensional structure of rRNA for evaluating sequence alignments of rRNA genes. In cases where one of the sequences has a known three-dimensional structure it can be more informative to compare the alignment with the solved structure, to better understand how the local environment of the nucleotides relates to conservation. In this regard, all-atom structure of ribosomal RNA of *Escherichia coli *[13] deduced from the crystal structure of 30S ribosomal subunit of *Thermus thermophilus *[11], can be used as a reference structure to evaluate individual rRNA sequences and the multiple alignments of rRNA genes. Thorough knowledge of the three-dimensional structure coupled with the secondary structure information of rRNA is often necessary to determine true evolutionary relationships among the rRNA sequences.

Furthermore, information derived from comparative rRNA sequence analysis has been extensively applied in microbial ecological studies. Presence of highly conserved and variable regions within the rRNA sequences is frequently used to identify oligonucleotide target regions unique to phylogenetic entities, for use as taxon-specific hybridization probes or PCR primers. The rRNA-targeted oligonucleotide probes have evolved into a widely used tool for the direct, cultivation-independent identification and enumeration of individual microbial cells or specific groups of bacteria in simple to complex natural environments. One of the hurdles in carrying out successful hybridization of rRNA sequences is the probe target site accessibility within the cell. The problems of target inaccessibility is often attributed to strong interactions of rRNA with ribosomal proteins and/or highly stable secondary and tertiary structure elements of the rRNA itself [14]. Thus, a thorough *in silico *evaluation of probe targets with respect to higher-order rRNA structures often is helpful, although the native structure of the ribosomes is altered by *in situ *fixation and hybridization procedures [3].

In this paper, we describe a program, RNA3D, developed using OpenGL to visualize and evaluate three-dimensional structure of 16S rRNA molecule and alignments of rRNA sequences, respectively. The program is capable of merging structural information with the phylogenetic or any other information derived from the sequence alignments, dynamically. The integration into the ARB software package [15] achieves interoperability among the various tools extending the functionality of ARB software suite substantially.

## Implementation

### Interface

The RNA3D program uses the popular OpenGL graphics library combined with Open Motif user interface for achieving more intuitive rendering and manipulation of the rRNA molecule with in the ARB environment. The annotation of RNA three-dimensional structures consists of a preprocessing of the information embedded in their 3D coordinates. It processes PDB structural information stored in the PDB file (1M5G) into the annotated structures and renders them into the virtual space using OpenGL routines. In order to objectively represent the structural knowledge of three-dimensional rRNA structure, the respective 3D coordinates were extracted from the PDB file (1M5G) and used for further structural analysis and searches. To provide user with a more detailed perspective of 16S rRNA structure, structural information corresponding to the ribosomal proteins were excluded during processing. The extracted structural information is then fed to OpenGL engine, where it is further transformed into a hierarchy of OpenGL objects, which encode molecule chains, residues and base positions. At this stage, further processing may occur, for example when the user requests the mapping of secondary structure information of rRNA onto the molecule in the form of loops and stems. Any information derived from the multiple alignments (phylogenetic information) is merged into the structural information of rRNA molecule in the post-processing step.

To achieve more performance and dynamic overlay of any sequence associated information, rendering was simplified to chain display with a capacity to display the actual residues – Adenosine (A), Guanine (G), Cytosine (C) and Uracil (U) at the respective coordinates in the molecule. Most of the applications which are intended to display three-dimensional structures, display the entire chemical structure of the molecule. Viewing the entire chemical structure in the molecule's 3D structure is less readable for the user. Additionally, base positions can be displayed at the respective coordinates or at the intervals specified by the user.

### Navigation

The entire set of visualized objects can be easily rotated, translated and scaled at the user's wish. Navigation through the molecule is basically bound to the standard mouse buttons and mapped to simple keys on the keyboard. The molecule can be zoomed in or out by performing upward or downward motion of the wheel, respectively. By rotating, translating and scaling of the molecule users can observe the buried and exposed molecule sections. Furthermore, the current cursor position in the respective sequence of alignments visualized in the primary or secondary structure editor can be shown in the three-dimensional structure.

### Customization

Since the user customization is an important consideration in the graphical user interface (GUI) design, RNA3D program provides the individual users with more possibilities to customize the interface to suit their particular purpose and preferences. As a first step toward enhancing the user customization capability of RNA3D program, any form of annotation and information overlay can be toggled on and off. This feature allows users to focus on annotations they consider important without being distracted by the information irrelevant to their particular needs. Additionally, users are provided with more customization capabilities in the form of specifying different colors, shapes, letters, and size of the objects rendered onto the scene at any time using Color Palette, Bases, Helix, Molecule and Mapping buttons of the RNA3D program. For example, users can colorize the entire molecule based on the residues that are participating in the loop or stem formation in the accepted secondary structure model of 16S rRNA. By defining color range, users can generate more informative 3D structural maps of 16S rRNA from the overlay of sequence associated information.

### Interoperability

RNA3D program readily establishes connection with the underlying central ARB database and ARB probe server [15]. The program co-operates with other tools housed in the ARB software package such as primary and secondary structure editors [15], probe design and evaluation tools [16]. Any change in the data and co-operating tools will be automatically updated in the program.

### Sequence and structural data

The public release of curated small subunit rRNA database from the ARB project [17] was used as a source for rRNA data. The secondary structure models of small subunit rRNA used are according to the comparative RNA website [18]. The 30S ribosomal subunit structures of *Escherichia coli *(PDB entry 1M5G) and *Thermus thermophilus *(PDB entry 1J5E) are retrieved from the protein data bank [19] and used as template structures for the RNA3D program.

## Results and discussion

The structural information extracted from the PDB files is rendered in an OpenGL 3D environment to achieve a detailed three-dimensional structure of 16S ribosomal RNA. The rendering speed critically depends on the computational platform where systems highly optimized for OpenGL are at a greater advantage for their graphical performance. The RNA3D program is based on the tacit assumption that all the molecules within a family have a common core with respect to three-dimensional shape which is supported by a common secondary structure that allows key functional groups to adopt similar spatial positions. Thus, the atomic structure model of *E. coli *30S ribosomal subunit [13] is taken as a reference structure to evaluate rRNA sequences and is further substantiated with the availability of very few rRNA crystal structures. Furthermore, the studies conducted by Gutell and coworkers have confirmed the accuracy of the covariation-based secondary structure models of rRNA with the crystal structures of ribosomal subunits [10]. Such studies support the inclusion and usage of three-dimensional structures of rRNA for carrying out rRNA based studies.

### Merging secondary structural information

**Figure 1:**
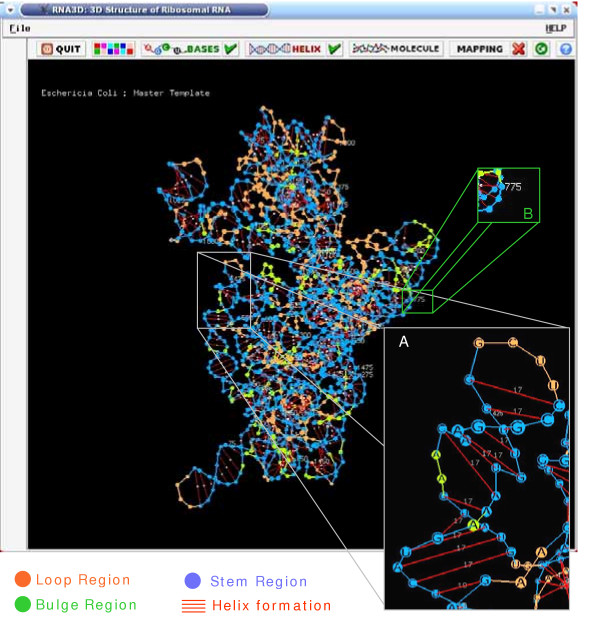
**Three-dimensional structure of 16S rRNA**; Screenshot showing rendered three-dimensional structure of small subunit ribosomal RNA in OpenGL 3D environment. In this screenshot, secondary structural features are combined with the three-dimensional structure of 16S rRNA. Residues representing loop regions and bulge regions of the 16S rRNA model are colored orange and green, respectively. And the residues participating in helix formation are colored blue. Part of the structure along with secondary structure interactions is shown in more detail in the inset (A). Letters A, G, C and U denote the actual residues in the 16S rRNA sequence. The numbers shown on the helices (colored red) represent the respective helix numbers in the secondary structure model. Respective nucleotide positions in the 16S rRNA sequence are displayed in grey (inset B).

The rRNA structural motifs (stems, bulges and loops) present in well established comparative structure models of rRNA [10] are extracted and merged with the three-dimensional structure data of small subunit rRNA (Figure 1). Furthermore, observations such as presence of intramolecular interactions (rRNA tertiary interactions) [20] in the loop regions of the structure can be evaluated with respect to three-dimensional conformations of ribosomal RNA in real time. The tertiary interactions are attributed to their role in stabilizing tertiary fold of rRNA [13] and hence they are highly conserved. The deeper insight into the rRNA crystal structure along with the secondary and tertiary interactions will have the potential to assist the user in refining the multiple sequence alignment itself when a large number of datasets is included.

### Mapping rRNA sequence data

**Figure 2:**
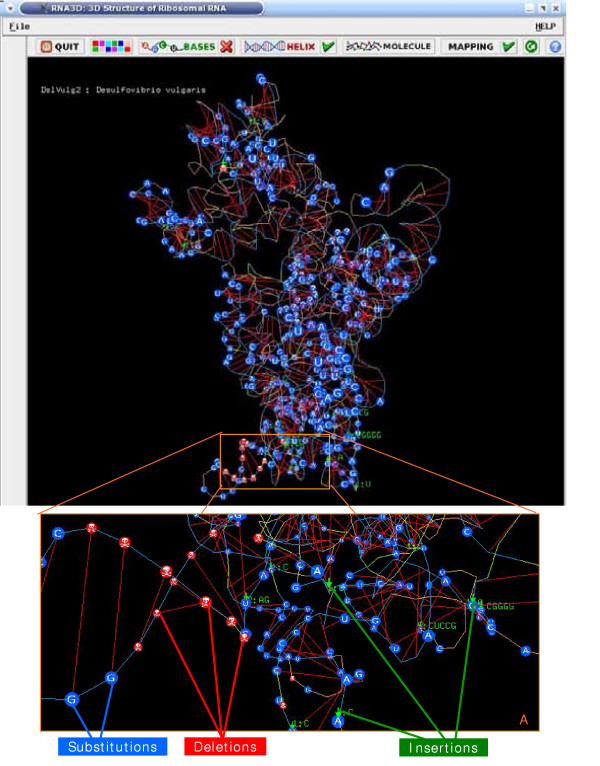
**Mapping rRNA sequence data onto 3D structure of 16S rRNA**; Screenshot displaying rRNA sequence data from *Desulfovibrio vulgaris *superimposed onto the 3D structure of *Escherichia coli *small subunit rRNA. The rRNA sequence of *D.vulgaris *is initially aligned in reference to *E.coli *rRNA sequence and then mapped onto the *E.coli *3D structure. Residues in blue represent substitutions or mutations where as regions in red denotes deletions in reference to *E.coli *rRNA sequence. Green arrows with residues indicate the positions and number of plausible insertions. Part of the structure is shown in more detail in the inset (A). Together with the positional variability maps (Figure 3) specific regions of individual rRNA sequences can be inspected thoroughly residue-by-residue.

Principally, any 16S rRNA sequence can be mapped onto the three-dimensional structure of small subunit rRNA. The RNA3D program swiftly performs a pair-wise comparison of a sequence selected in a multiple alignment using *E.coli *as a reference and maps it onto the rRNA 3D structure. The selected rRNA sequence is annotated with mutation (base substitutions), insertion and deletion information at each site as compared to the master sequence (*E. coli*). For the regions where the sequences are aligned without deletion or insertion, direct base substitution (mutation) is applied. Because the C'---C' distance is essentially the same (~ 10.2 Å) in all Watson-Crick base pairs [22], this simple procedure preserves the base pairing and the double helical structure while substituting the bases. Although there do exist the requirement of structural adjustments for non-Watson-Crick base pairs, currently, simple base substitutions are kept because the development of new models to achieve the necessary structural adjustments is out of the scope of the RNA3D program. In the regions where the alignment (of selected rRNA sequence) involves insertions, the respective insertion points corresponding to *E. coli *base position in the alignment are shown as down arrows in the crystal structure (Figure 2). The number of insertions and the participating nucleotides can also be displayed at the insertion points. In the case of regions where deletions are observed in the alignment corresponding to the master sequence (*E. coli*), respective sites in the 3D structure are indicated as deleted, using  symbol (Figure 2). At present, the program displays the deletion and insertions in the 3D model in reference to *E.coli*. The structural implications of such deletions and insertions are not handled by the program because all the coordinates of the model were experimentally determined not modeled. The program only highlights the sites of deletion and insertion points in the 3D model. In future, as more and more RNA crystals become available in PDB, users will have the possibility to switch the 3D models, dynamically, to closely related organism rather than referencing to *E.coli*, to minimize/eliminate the deletion and insertion sites in the 3D model.

Overlaying of mutation, deletion and insertion information at each site of the sequence alignment when coupled with the secondary and tertiary interactions of rRNA, gives the user an over all view of the individual rRNA sequences with respect to the resolved crystal structure (Figure 2). Since the accuracy of the phylogenetic tree is dependent on the proper juxtapositioning of the sequences in the alignment [10], RNA3D program enables the user to approximate the best juxtapositioning of sequences that represent similar placement of nucleotides in their fitted structural conformation with respect to the master structure. When coupled with ARB secondary structure editor [15], more accuracy can be achieved in aligning diverse rRNA sequences. Sequences that form the same secondary and tertiary structure can be juxtaposed by aligning the positions that form the same components of the similar structural elements (for example, aligning the positions that form the base of the helix or the hairpin loop). Additionally, the entire sequence cannot be viewed at once in primary sequence alignments, so by superimposing the sequence onto the 3D structure the user can get a complete view on the entire sequence. The secondary structure models of rRNA were basically developed based on the comparative paradigms that the different RNA sequences can fold into the same secondary and tertiary structures and the unique structure and function of RNA molecule are maintained through the evolutionary process of mutation and selection [23,24]. The same assumption can be extended to the three-dimensional structures of rRNA as there are, at present, very few rRNA crystal structures deposited in the protein data bank.

### Overlaying information derived from sequence alignments

**Figure 3:**
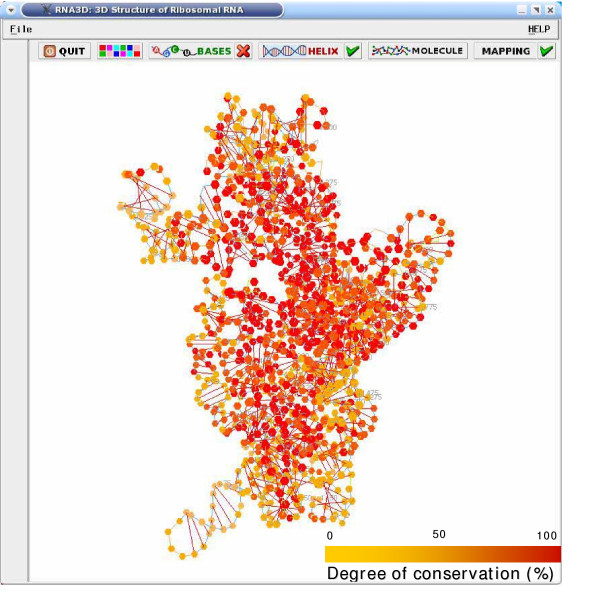
**Conservation profile of 16S rRNA three-dimensional structure**; Screenshot showing the conservation profile or positional variability information superimposed onto the 3D structure of small subunit rRNA. Column statistics are performed on the multiple alignments using parsimony method and minimum number of mutations for each site is determined. The positional variability values are then overlaid onto the 3D structure of 16S rRNA of *Escherichia coli *residue-by-residue to generate 3D positional variability maps. Residues inclining towards yellow are highly variable where as the residues inclining towards red are highly conserved positions. It is apparent that the highly variable residues are located on the periphery of the 3D structure while the highly conserved residues are centralized at the core of the structure. Conservation profile was calculated with the data set containing around 40,000 complete 16S rRNA sequences.

Dynamic overlay of information derived from the underlying sequence alignment onto the molecule enables the users to observe any sequence derived meta-information at the individual residues in a three-dimensional spatial environment. When variability maps are overlaid onto the structure, users can identify the conserved and variable regions in the small subunit of the ribosomal RNA. Sequence variation with respect to the loop and stem regions of the rRNA structure can be seen when mutation information (calculated for the overall sequences in the database) is superimposed. By setting specific colors to rRNA structural motifs and interactions, one can immediately see the differences and distribution of potential interactions in the small subunit of rRNA 3D structure. Additionally, intermolecular contacts i.e., between 16S rRNA bases and the ribosomal protein residues, which are important to stabilize the tertiary fold of the rRNA, as well as the complex formation of the ribosome [21], can be visualized in the crystal structure. Simple to complex column statistics that are performed on the multiple alignments of rRNA genes can also be readily overlaid onto the 3D structure of rRNA. Column statistics such as sequence consensus, base frequency, positional variability based on parsimony method are calculated using the integrated tools of ARB package [15]. The column statistics are transformed into different colors based on the user-defined rules and more informative three-dimensional structural maps of rRNA are generated (Figure 3). Superimposition of such statistical data (e.g. positional variability, base frequency) or information derived from the sequence alignments aids the users to carry out in-depth evaluation of multiple sequence alignments.

### Structural evaluation of rRNA targeted probes

**Figure 4:**
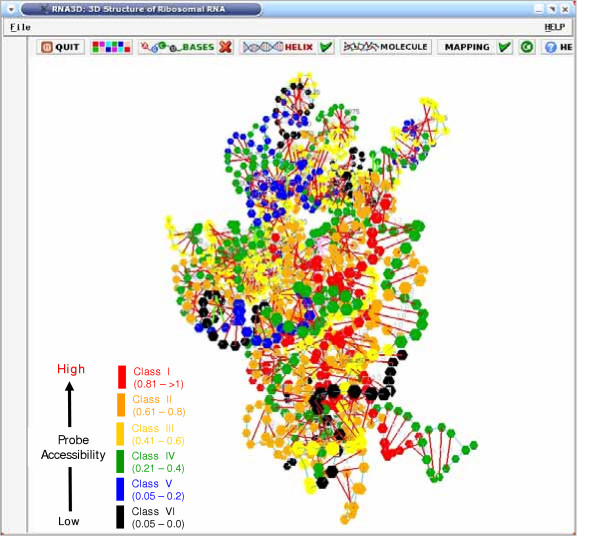
**Probe accessibility map of 16S rRNA three-dimensional structure**; Screenshot showing the distribution of relative fluorescence hybridization intensities of oligonucleotide probes targeting 16S rRNA of *Escherichia coli*. Probe accessibility data is according to Behrens et al [25]. Probe accessibility information is mapped onto the 3D structure of *E.coli *16S rRNA. The different background colors indicate the brightness range of different classes (classes I through VI) with respect to the observed fluorescence intensities. Residues colored red are readily accessible where as residues colored black are virtually inaccessible for the rRNA targeted probes. Such 3D accessibility maps can be used to evaluate oligonucleotide probes with respect to 3D conformations of rRNA, when intended to be used in FISH experiments.

Oligonucleotide probes targeting small subunit rRNA are frequently applied in the molecular ecological studies employing the technique of Fluorescence *in situ *hybridization. Since the hybridization is influenced by the higher-order rRNA structures [14], the structural features exhibited by rRNA should be considered during the design and evaluation of rRNA targeted probes. Structural conformations of target and neighboring regions of rRNA are crucial for hybridization behavior and hybrid stability. Studies have shown that the differences in higher-order structures of rRNA do have considerable influence on the target site accessibility to rRNA targeted probes even though the small subunit rRNA is a highly conserved molecule [25]. In accordance, accessibility studies on 16S and 23S rRNA of *E. coli *and other organisms with respect to FISH experiments revealed that some regions of *E. coli *ribosome are virtually inaccessible for oligonucleotide probes when FISH is performed [25-28]. Availability of accessibility data on members of the domains *Bacteria*, *Archaea *and *Eukarya *led to the development of consensus models for the accessibility of the small subunit rRNA to oligonucleotide probes [25]. *In silico *evaluation of rRNA targeted probes with respect to the consensus probe accessibility models and rRNA secondary structure has been recently reported [16]. Using RNA3D program, users can get more insights into the proposed probe candidates with respect to three-dimensional conformations of small subunit rRNA. The localization of the targets of single or multiple [29] probes can be visualized simultaneously in customizable background colors with in the rRNA 3D structure. By adjusting the zoom level and rotating the molecule, users can get an idea about the probable binding site of the proposed probe with respect to the structural conformation of rRNA. Considering both secondary and tertiary structural interactions of rRNA target users have an opportunity to evaluate the probe targets with more confidence before making any decision on the selection of probes. Although the native conformation of ribosomes is disturbed during the experimental procedures of FISH [25], a thorough *in silico *evaluation of oligonucleotide probes with respect to the higher-order structure and experimental accessibility data (Figure 4) may help the users to design more successful hybridization experiments.

### Related work

Several programs have been developed in recent years in order to achieve overlaying of information derived from the multiple alignments onto the three-dimensional structures [30,31]. Most of the programs are limited to static displays and are restricted to protein molecules. A somewhat flexible system with dynamic capabilities to visualize 3D structures has been recently developed [32]. With respect to sequence alignment evaluation, the ARB facility of direct cooperation of the respective tools and the alignment editor is missing in such systems. Furthermore, none of the programs mentioned does support the superimposition of oligonucleotide probes and any additional data that is associated with rRNA genes onto the rRNA 3D structure. Such unique features of RNA3D program are seldom found in the existing tools, which are more specialized to visualize the molecules deposited in the protein data bank. In this regard, our program, RNA3D, with its dynamic capabilities operating together with the several tools of ARB package, offers a special platform to carry out in-depth structural analysis with respect to ribosomal RNA.

Since the RNA3D program uses OpenGL with dedicated graphics hardware, the processing capabilities offered by such graphics cards (known as Graphics Processing Units – GPU) can be utilized for accelerating the program in future. Using GPUs as coprocessors, non-graphic computations can be performed speeding up the performance of the applications significantly which is useful for further extension of RNA3D program (see General Purpose Computation on Graphics Processing Units – GPGPU [33]).

## Conclusion

Visualization of three-dimensional structure of ribosomal RNA in an intuitive display provides the biologists with the greater possibilities to carry out structure based phylogenetic analysis. The RNA3D program allows the changing of display parameters while the molecule is being displayed without compromising with the performance. This is very important to observe any inference drawn with the underlying sequences in the real-time environment. Mapping individual rRNA sequence onto the template structure, users can visually inspect the quality of the local alignment and identify the regions that may need any manual checking for further refinement of sequence alignments. By superimposing column statistics or information derived from the sequence alignments onto the rRNA 3D structure, users can get more insights into the individual rRNA genes and carry out in-depth evaluation of multiple sequence alignments. Dynamic overlay of information derived from the underlying sequence alignment onto the molecule enables users to observe any sequence inherited characteristics (phylogenetic and other information) that influence the individual residues in a three-dimensional virtual environment. With the possibility of visualizing oligonucleotide probes and mapping probe accessibility models, users can virtually observe the secondary and tertiary structural implications of ribosomal RNA on the prospective probe *in silico*. This feature might serve as valuable information during designing successful *in situ *hybridization experiments. The integration of RNA3D program into the powerful and widely used ARB software package enables the communication with the several tools of ARB package achieving interoperability. Therefore, along with the other tools of ARB, RNA3D offers the researchers with an all-in-one software platform to carry out a thorough sequence analysis with much deeper perspective, which is seldom found to their disposal. In the future, programs with 3D environments will become more important as tools for bioinformatics, as they provide much higher possibilities to integrate molecular sequence data, structure data and analysis data on one platform.

## Availability and requirements

The binaries and source code of the program can be freely downloaded along with the ARB software package from our project website [17]. The up-to-date, aligned and annotated ribosomal RNA databases are also made freely available for the scientific community. Probe accessibility models and other structure data used in the program to demonstrate can be obtained by requesting the authors. Currently, the ARB software is available for PCs running LINUX operating systems and SUN SOLARIS systems.

## Authors' contributions

YK developed and implemented the tool and drafted the manuscript. RW participated in design and implementation. PK offered technical help during the development. WL initiated the development of the tool and supervised the ARB project. All the authors read and approved the manuscript.
